# The Prognostic Value of Methylation Signatures and *NF2* Mutations in Atypical Meningiomas

**DOI:** 10.3390/cancers13061262

**Published:** 2021-03-12

**Authors:** Rahmina Meta, Henning B. Boldt, Bjarne W. Kristensen, Felix Sahm, Wenche Sjursen, Sverre H. Torp

**Affiliations:** 1 Department of Clinical and Molecular Medicine, Faculty of Medicine and Health Sciences, Norwegian University of Science and Technology (NTNU), 7491 Trondheim, Norway; wenche.sjursen@ntnu.no (W.S.); sverre.torp@ntnu.no (S.H.T.); 2 Department of Pathology, Odense University Hospital, 5000 Odense, Denmark; Henning.Boldt@rsyd.dk (H.B.B.); bjarne.winther.kristensen.01@regionh.dk (B.W.K.); 3 Research Unit of Pathology, Department of Clinical Research, University of Southern Denmark, 5000 Odense, Denmark; 4 Department of Pathology, The Bartholin Institute, Rigshospitalet, Copenhagen University Hospital, 2100 Copenhagen, Denmark; 5 Biotech Research and Innovation Center (BRIC) and Department of Clinical Medicine, Faculty of Health and Medical Sciences, University of Copenhagen, 1165 Copenhagen, Denmark; 6 Department of Oncology, Odense University Hospital, 5000 Odense, Denmark; 7 Department of Neuropathology, Institute of Pathology, University Hospital Heidelberg, 69120 Heidelberg, Germany; felix.sahm@med.uni-heidelberg.de; 8 German Cancer Research Centre CCU Neuropathology (DKFZ), 69120 Heidelberg, Germany; 9 Department of Medical Genetics, St. Olavs Hospital, 7030 Trondheim, Norway; 10 Department of Pathology, St. Olavs Hospital, 7030 Trondheim, Norway

**Keywords:** brain tumors, meningiomas, prognosis, diagnosis, methylation profiling, molecular genetics

## Abstract

**Simple Summary:**

The WHO 2016 classification of human meningiomas is debated due to the subjective evaluation of the histopathological diagnostics and grading. However, meningioma classification based on genome-wide DNA methylation profiling has become useful in classification of these tumors by being a better prognostic tool. The current pilot study was designed to test out genome-wide DNA methylation profiling on atypical meningiomas as these tumors have a highly variable risk of recurrence. Although we found that it had diagnostic value, further refinements on the methylation profile procedure are required. With this study we aim to motivate and impact researchers to continue to work and debate towards an improved meningioma classification including molecular and genetic biomarkers, which will benefit patients with such diagnoses.

**Abstract:**

*Background*: Due to the solely subjective histopathological assessment, the WHO 2016 classification of human meningiomas is subject to interobserver variation. Consequently, the need for more reliable and objective markers are highly needed. The aim of this pilot study was to apply genome-wide DNA methylation analysis on a series of atypical meningiomas to evaluate the practical utility of this approach, examine whether prognostic subclasses are achieved and investigate whether there is an association between the methylation subclasses with poor prognosis and time to recurrence. *NF1/2* mutation analyses were also performed to explore the prognostic value of such mutations in these atypical meningiomas. *Methods*: Twenty intracranial WHO grade II atypical meningiomas from adult patients were included. They consisted of 10 cases with recurrence (group I), and 10 cases without recurrence (*group II*). The formalin-fixed and paraffin-embedded tissues underwent standardized genome-wide DNA methylation analysis, and the profiles were matched with the reference library and tumor classifier from Heidelberg. *NF1/2* somatic mutation analyses were performed using the CNSv1panel from Düsseldorf. *Results*: Eighteen out of 20 cases matched to the meningioma class using the common brain tumor classifier (v11b4). Four of these cases matched to a methylation subclass related to a prognostic subgroup based on a cut-off of 0.9. *NF2* mutations were detected in 55% of cases across both groups, and the most prominent copy number alterations were chromosomal losses of 22q, 1p and 14q. No significant *NF1* mutations were identified. *Conclusions*: Genome-wide DNA methylation profiling represents a useful tool in the diagnostics of meningiomas, however, methodological adjustments need to be addressed.

## 1. Introduction

Meningiomas are a group of mostly benign, slow-growing neoplasms [1,2]. They are the most frequently reported primary intracranial tumor, accounting for 37.6% of brain tumors overall [3]. These neoplasms are classified by various histopathological criteria given in the WHO (World Health Organization) classification of 2016 CNS (central nervous system) tumors [4]. They are divided into three major histopathological groups reflecting their general malignancy; grade I equals to benign, grade II as atypical (intermediate), and grade III as malignant (anaplastic) [4]. The current classification system for meningiomas has shown to hold a high degree of inter-rater concordance [5]. However, since the classification is solely based on subjective morphological criteria, the reproducibility still remains suboptimal which in consequence makes the prediction of prognosis and planning of follow-up schemes challenging [6,7,8]. Up to 25% of benign meningiomas predicted to give a benign clinical course recur, while up to 71% of atypical meningiomas predicted as more malignant do not recur [4]. Atypical meningiomas in particular show a wide range of variable clinical behavior, which might be explained by the heterogeneity of this tumor type [6,9]. Therefore, more robust and objective tools are needed to be incorporated into the future pathological classification of meningiomas to enhance both the diagnosis, the prognosis, and the patient management.

The application of multiplatform genomic and epigenetic investigations has led to a paradigm shift over the past few years in the research of meningiomas, beneficial with regard to postoperative patient management. There has been, for instance, identified a unique gene expression signature that more accurately stratifies patients with meningioma with respect to recurrence risk [10]. Regarding cytogenetic and genetic findings, loss of chromosome 22q, deletion of chromosome 1p and *NF2* (neurofibromatosis type 2) mutations are commonly encountered [11,12,13]. Furthermore, mutations in *TERT* (telomerase reverse transcriptase) promoter and loss of histone H3K27me3 have been associated with increased risk of recurrence [14,15,16]. Moreover, the entire WHO meningioma scheme from 2016 has been challenged by introducing a classification based on genome-wide DNA methylation profiling. This classification has shown to be more precise in predicting patients at high risk for disease progression, tumor recurrence and prognosis than the current WHO classification [17].

According to the study of Sahm et al., WHO grade II atypical meningiomas mainly attained prognostic methylation subclasses as benign-1, intermediate-A and intermediate-B [17]. Based on these findings, the aim of this pilot study was to examine whether our series of atypical meningiomas would achieve such prognostic subclasses, investigate whether there is an association between the prognostic methylation subclasses with poor prognosis and TTR (time to recurrence), and evaluate the practical approach of genome-wide DNA methylation profiling on human meningiomas. We also wanted to explore the prognostic value of *NF1/2* mutations in these WHO grade II atypical meningiomas.

## 2. Materials and Methods

### 2.1. Material Collection

Twenty atypical meningiomas from 1991 to 2006 were investigated. The meningiomas were retrospectively reviewed and included by applying the histopathological definitions and systematic approach described in a previous protocol [18]. Maximum follow-up time was 18 years. All patients underwent surgery for primary meningiomas at St. Olavs University Hospital, Trondheim, Norway. The selected cases were found by searching in the electronic patient files at Department of Pathology. Relevant medical and clinical information was collected from medical records at St. Olav University Hospital and local hospitals.

**Figure 1:**
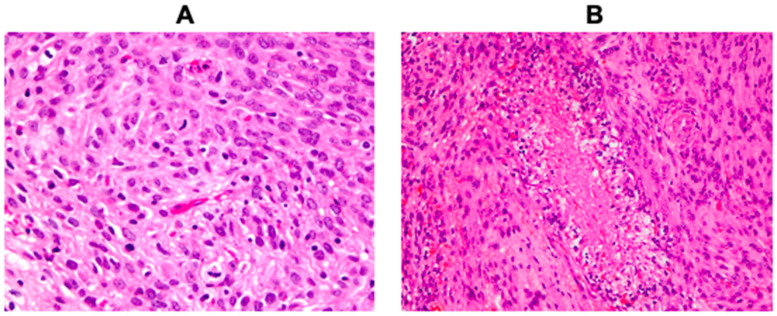
Histopathology of atypical meningiomas. (**A**) An atypical meningioma with several mitoses (40× objective). (**B**) An atypical meningioma with hypercellularity and necrosis (20× objective).

Inclusion criteria were primary intracranial atypical meningiomas graded according to WHO 2016 classification based on mitotic counts or atypical features (with at least three of the following histopathological features; small cells with high nuclear-to-cytoplasmic ratio, increased cellularity, prominent nucleoli, sheeting and spontaneous foci of necrosis) (Figure 1) [4]. All included cases were immunoreactive for EMA (epithelial membrane antigen) for verification of the meningioma diagnosis. Exclusion criteria were patients < 18 years, brain-invasive meningiomas, cases with congenital *NF1/2* mutations, clear cell and chordoid meningiomas, cases with Simpson resection grade greater than III and patients who received postoperative radiation.

The clinical information collected from patient medical files were date and age at surgery, gender, tumor localization, treatment given, Simpson resection grade, date of recurrence, last observation of the patient and cause and date of death. TTR was defined as tumor related death from the time of diagnosis, or the time from date of surgery to date of tumor recurrence or tumor progression verified either by MRI or CT [19]. Survival data was collected from the Norwegian Cause of Death Registry and patient medical files.

**Figure 2:**
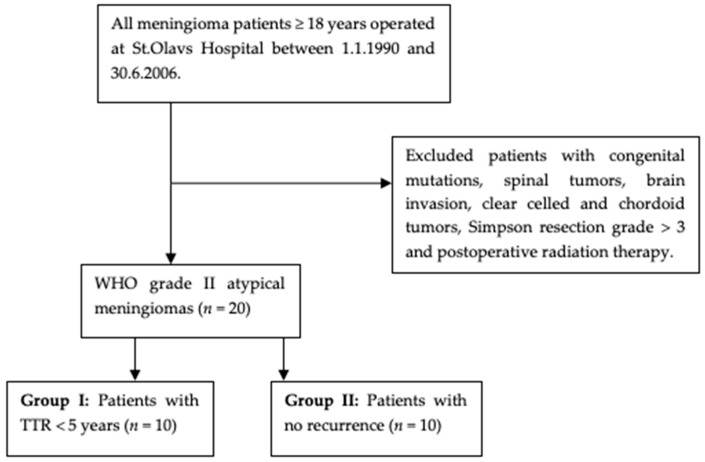
Flowchart of patient selection.

The atypical meningiomas were divided into two groups: Group I included 10 cases with TTR < 5 years, and Group II included 10 cases with no recurrence. These two groups of WHO grade II atypical meningiomas graded by similar histopathological criteria were thought to demonstrate associations between their recurrence rates and their respective methylation profiles. Figure 2 illustrates the selection of meningioma cases.

### 2.2. DNA Purification

DNA was purified from 10 µm paraffin slides using GeneRead DNA FFPE Kit (Qiagen, Hilden, Germany) according to the manufacturer’s instructions.

### 2.3. Next-Generation Sequencing

Ion AmpliSeq CNS NGS (Next-Generation Sequencing) Panel v1 (CNSv1-NGS) is a glioma-targeted custom designed gene panel, covering the entire coding sequences or hotspot regions of 20 genes frequently mutated in brain tumors, including *NF1* and *NF2*. The panel targets 66,418 bases in 660 amplicons and has been described in detail previously [20,21]. DNA was quantified using an RNase P TaqMan Copy Number Reference Assay performed on a QuantStudio 12K Flex Real-Time PCR System (Applied Biosystems, Foster City, CA, USA). NGS libraries were prepared in two primer pools using the Ion AmpliSeq Library Kit 2.0 containing Ion AmpliSeq HiFi Mix and Ion AmpliSeq CNSv1-NGS gene panel primer pool in 10 µL reaction volume with 5 ng genomic DNA per primer pool as template. All Ion products were supplied by Thermo Fisher Scientific, Waltham, MA, USA. Barcoding and adapter ligation of the amplified DNA was performed with the Ion Xpress Barcode Adapters 1–16 Kit according to the manufacturer’s instructions. Library quantitation was performed using the Ion Library Quantitation Kit. Emulsion PCR and sample preparation was performed using the Ion PGM Hi-Q View OT2 Kit on the Ion OneTouch 2 and Ion OneTouch ES instruments. Sequencing was performed using the Ion Torrent PGM System with Ion PGM Hi-Q View Sequencing Kit and Ion 318 Chip Kits v2, resulting in 400,000–600,000 mapped reads per sample. A tonsil control sample from a healthy donor was included in all sequencing experiments. Data analysis, including base calling, quality scoring, trimming, demultiplexing, and alignment, was performed using standard Ion Torrent Suite workflows (software versions 5.0 through 5.8, Thermo Fisher Scientific, Waltham, MA, USA) and GRCh37/hg19 as human reference genome. Plugins were coverageAnalysis, IonReporterUploader and variantCaller. NGS data was analyzed for sequence variants using Ion Reporter v5.10 (Thermo Fisher Scientific, Waltham, MA, USA) with a filter chain removing variants annotated as single-nucleotide polymorphisms (SNPs) by the UCSC Common SNPs database and intron variants, except for splice site positions. BAM alignment files were manually analyzed for DNA sequence aberrations using Golden Helix GenomeBrowse 2.1.0 (Golden Helix, Bozeman, MT, USA).

### 2.4. Copy Number Variation Detection Based on NGS Panel Data and Illumina Infinium Methylation EPIC Bead Chip Array Analysis

CNV (copy number variation) was detected using Ion Reporter v5.12 software based on a Hidden Markov Model algorithm that uses normalized read coverage across amplicons to predict the copy number or ploidy states. CNV analysis was performed with a workflow based on a custom copy number baseline consisting of more than 40 tonsil control samples including 10 biological replicates, and six glioma tumor samples with no recognizable CNVs by 850k methylation profiling.

EPIC bead chip array analysis was performed to collect data on the DNA methylation status of >850,000 CpG sites as previously described [22]. In brief, DNA quantitation was performed on Qubit 2.0 with Qubit^®^ dsDNA HS Assay (Thermo Fisher Scientific, Waltham, MA, USA). DNA quality was assessed by qPCR with SYBRGreen PCR Master Mix on QuantStudio 12K Flex Real-Time PCR System (Applied Biosystems, Foster City, CA, USA) based on the FFPE QC Kit from Illumina. Bisulfite conversion was performed using Zymo EZ DNA Methylation kit (Zymo Research, Irvine, CA, USA). The bisulfite converted DNA was restored using Infinium HD FFPE Restore Kit from Illumina and processed with ZR-96 DNA Clean & Concentrator-5 kit from Zymo Research. The EPIC bead chip array was prepared according to the manufacturer’s instructions and data collected with the iScan from Illumina. The EPIC bead chip array and associated reagents and solutions were from Illumina, San Diego, CA, USA.

Brain tumor classification and generation of CNV plot was performed in silico using the MolecularNeuropathology.org server (https://www.molecularneuropathology.org/mnp) using versions v11b4 for brain tumor classification and MNGv2.4 for meningioma subtype classification, respectively, as described by Sahm et al. and Capper et al. [17,23,24].

## 3. Results

Most patients were women with a female: male ratio of 2.3. The median ages at surgery were 68 (ranging from 36–86) and 62.5 (ranging from 38–83) years, in Group I and Group II, respectively, while the median age in total was 65 (ranging from 36–86) years. The tumors were located in the convexity in 80% of cases. Mean TTR in group I was calculated to be 18.3 months.

In overall, 90% of the cases matched to the meningioma class using the methylation ‘classifier’, and thus recognized as a meningioma. Two cases achieved neither a match in the ‘classifier’ nor a subclassification and thus not recognized as a meningioma. Based on cut-off value over 0.9, only four cases reached a subclassification in addition to a match in the meningioma ‘classifier’. One female (case 6) in Group I, with TTR of 42.0 months, achieved a subclassification as benign-1. In Group II, two males (case 17 and 19) achieved subclassifications as intermediate-A and intermediate-B, respectively, and one female (case 16) attained a methylation subclass as benign-1. By lowering the cut-off score to 0.6 (proposed by *Felix Sahm, pers. comm.*), more cases were subclassified with respect to a prognostic subgroup (Table 1). The exact score values for each case are listed in Supplementary Materials.

**Figure 3:**
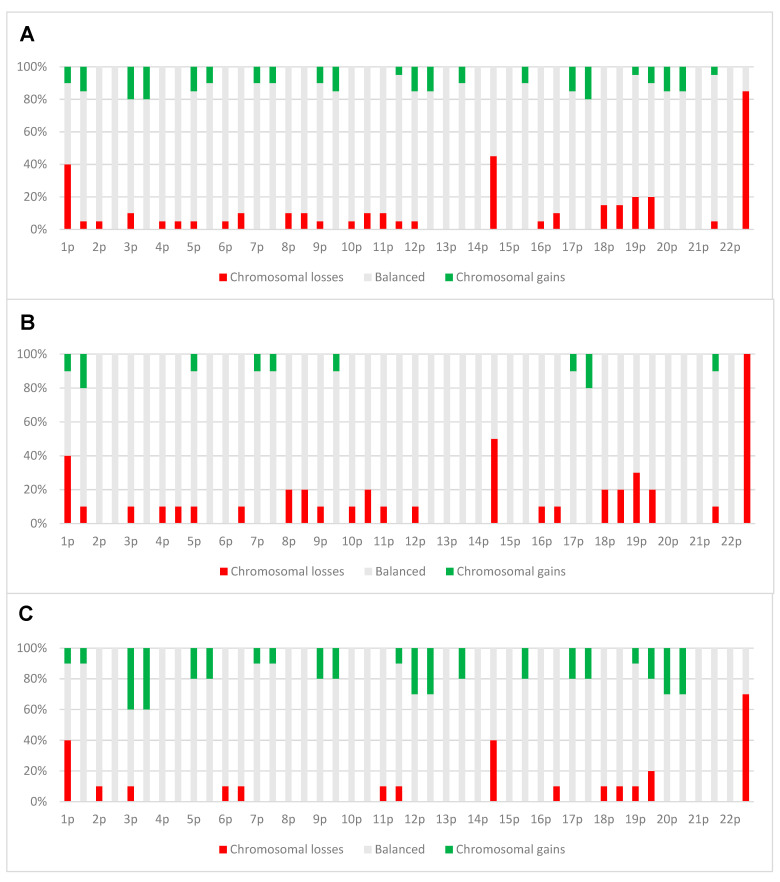
CNV profiles from methylation profiling data of all 20 cases (**A**) and profiles of cases distributed on group I (**B**) and group II (**C**), respectively.

The CNVs showed great variability, however, recurrent chromosomal losses in 1p (40%), 14q (45%) and 22q (85%) were observed (Figure 3A). Chromosomal losses were more frequent per case than chromosomal gains. Comparing the two prognostic groups, chromosomal losses were more frequent in Group I than in Group II and vice versa for chromosomal gains (Figure 3B,C).

The CNV profiles derived from the NGS data were far less sensitive to detect the CNVs in the cases compared to the 850k data due to limitations in coverage of panel sequencing. Yet, 22q loss based on coverage of *NF2* was estimated in 75% of cases by NGS, supporting the findings on 22q status from the 850k data.

*NF2* mutations were detected in 55% of cases; 70% of cases in Group I, and 40% of cases in Group II (Table 2). The mutations included frameshift, nonsense and splice site alterations, all interpreted to cause loss of NF2 protein function. The *NF2* gene resides at chromosome 22, and notably, all cases with *NF2* mutations had also losses in chromosome 22. Fifty percent of the cases were located in the convexities and had concurrent *NF2* mutations, while both skull base meningiomas did not have *NF2* mutations. *NF1* mutations were detected in two cases, one in each group being substitutions of one (exon 26) and two amino acids (exon 57), respectively. These variants are not reported in databases, making it difficult to interpret whether they have any functional effects at the protein level and determine their pathological significance. They are variants of uncertain significance (VUS) and therefore not attached any importance to.

## 4. Discussion

In the present pilot study, a series of atypical WHO grade II meningiomas underwent genome-wide DNA methylation analysis to evaluate the practical use of the technique, examine whether prognostic subclasses are achieved and investigate whether there is an association between the methylation subgroups with poor prognosis and TTR. We found that most cases attained a methylation pattern coinciding with the meningioma methylation class, recognizing the tumor samples as a meningioma. However, only a few samples achieved a subclassification associated with prognosis based on a cut-off value of 0.9. This study also explored cytogenetic and molecular genetic alterations, which revealed mainly copy number aberrations in terms of 1p, 14q and 22q losses, and *NF1/2* mutations in 65% of cases overall.

### 4.1. Genome-Wide DNA Methylation Analysis

As most cases (90%) attained a match in accordance with the meningioma “classifier”, it coincides adequately with the study from Sahm et al., registering that their DNA methylation data segregated meningiomas from other intracranial tumors [17]. Consequently, this is of diagnostic value as meningiomas have numerous differential diagnoses, making genome-wide DNA methylation profiling applicable when in question of this matter [8,17]. For case 5 and 13 that did not match with the meningioma “classifier”, we believe that this can be explained in brief by both biological and technical aspects of the methylation procedure (see Supplementary Materials).

Sahm et al. also showed that atypical meningiomas fall into subgroups (mainly methylation class benign-1, intermediate-A and intermediate-B) [17]. Yet, such subclassification was limited to only 20% of the samples in the present study when using a cut-off value of 0.9. This low number of subclassified tumor samples may be explained by various technical factors. In most cases the bisulfite conversion was problematic, and the frequency of measured CpG-sites were low, reflecting poor DNA quality in the FFPE (formalin-fixed, paraffin-embedded) samples. From previous studies, formaldehyde, the main component of formalin, is known to induce various histone-DNA, DNA-amino acids and DNA-DNA-crosslinks, resulting in a conformational change and partial denaturation of the nucleic acid [25]. In addition, the DNA quality may also have been affected by storage as the DNA fragments progressively with longer storage time [26]. According to the Heidelberg experience, old age of the FFPE-material does not preclude extraction of suitable DNA, whereas the quality and composition of the fixative appears to be more important [24]. Similar analysis carried out on old glioma samples have shown acceptable results, which could also indicate that the time of storage may have lesser impact on the DNA quality than the formalin fixation (*David Scheie, pers. comm.*). The fact that this study revealed few methylation subclasses reflects that methodologic adjustments in the approach of methylome profiling as a technique are required before implementation of it in the daily routine diagnostics of meningiomas. Similar conclusions have been drawn in the recommendations after the cIMPACT-Utrecht meeting [27].

Preserved genetic information in meningioma specimens is a prerequisite for molecular investigation as this is shown to give distinct epigenetic patterns and clinically relevant subgroups of meningiomas, enabling risk stratification of patients more accurately [28,29]. It is therefore important to establish routines that maintain the molecular genetics of the tumors. For instance, the use of fresh tumor tissue should be deliberated as a solution in this regard.

Adjustment of cut-off values may be considered [24]. The cut-off value applicable for matching prognostic methylation profiles was proposed to be lowered from 0.9 (used in the current MNG classifier version 2.4) to 0.6 in the current study. This resulted in more subclassified cases showing a weak trend towards enrichment of intermediate and malignant methylation profiles in group I and benign methylation profiles in *group II*. Which cut-off value that should be implemented, needs further evaluation, and in that regard, scores between 0.3 and 0.9 have been mentioned [24].

To our knowledge, there are few studies that have looked into the practical use of methylation profiling of human meningiomas. Thus, we are unaware of any available public databases for linkage of data to strengthen our results. However, in the study of Nassiri et al. there was implemented methylation data and clinical factors to estimate risk of recurrence [29].

Even though molecular genetical analyses such as NGS and methylation profiling are about to be implemented in various human malignancies, including tumors of the central nervous system, these analyses cannot yet be carried out at an affordable cost [30]. However, as such aforementioned techniques will be more refined and commonly used in the clinical diagnostics, it will allow better availability and cost-effectiveness.

### 4.2. Copy Number Alterations

The copy number alteration analyses conducted in the present study disclosed frequent chromosomal aberrations in both prognostic groups (Figure 3A), commonly encountered in more aggressive meningiomas [2,12]. Loss of chromosome 22 is the most frequent somatic copy number aberration reported for meningiomas [12]. This is followed by loss of chromosome 1p and chromosome 14q [31]. Equivalent findings are observed in the current study where chromosomal losses occurred predominantly in 22q, succeeded by chromosomal losses in 14q and 1p, respectively.

The MethylationEPIC 850k CNV analysis captured more chromosomal aberrations compared to the NGS technique due to higher sensitivity in the 850k MethylationEPIC CNV analysis. Similar changes have been found in both techniques, noting especially the comparable frequency of chromosomal aberrations in chromosome 22q.

Chromosomal aberrations have in some studies been shown to be of prognostic value in meningiomas [31,32,33]. However, in the study of Nassiri et al. they found that such alterations were not significantly associated with recurrence [29]. In the present study, there is a tendency of more chromosomal losses in Group I compared to Group II, and a tendency of more chromosomal gains in Group II compared to Group I (Figure 3B,C). This may indicate that a higher rate of chromosomal losses can be associated with a poorer prognosis, while a higher rate of chromosomal gains could act as a protective factor against recurrence in atypical meningiomas. However, these findings are contradictory to previous studies, which suggest that there is more genetic instability and complex karyotypes, both losses and gains, with increasing malignancy grade in meningiomas [2,12,31,34]. This should therefore be investigated in a larger sample cohort with suitable statistical methods.

### 4.3. NF1/2 Mutations

Frequent *NF2* mutations were observed in this series of atypical meningiomas across both groups, yet more prominent in Group I. Previous studies reported that *NF2* was mutated in about half of the cases, whilst the present study found that 55% of the samples were mutated [11,31]. In addition, mutations in the *NF2* and losses in chromosome 22 co-occurred in all cases in the present study, which is also found in previous studies, although to a varying extent [11,31].

Furthermore, the *NF2* mutations are preferentially located in meningiomas at the convexities, whilst skull base tumors harbor other mutations and hence driven by other genetic alterations [35]. In the current study, we found that *NF2* mutations in the convexities occurred in 50% of samples, and *NF1* mutation occurred in one case located in the skull base (basal). Case 18, with tumor in the basal, had neither of the mutations, and could be driven by other mutations commonly found in meningiomas from the skull base [35].

### 4.4. Strengths and Limitations

A strength of this study is the long follow-up time on patients with complete clinical data that made it possible to establish two groups of patients with significant different clinical outcome. In addition, it is also beneficial that all patients are from one treatment center with a population-based referral. Even though few cases were examined, this study sheds light on problematic issues regarding methylation analyses on FFPE meningioma samples that need to be solved. Limitations encountered are the usual nature of retrospective studies, the selection of cases that might provide with bias, few cases, as well as old FFPE-tissue samples which may be influential on DNA quality.

## 5. Conclusions

In conclusion, this study has demonstrated that meningioma methylation classification is applicable in routine diagnostics, which can be valuable in cases with difficult differential diagnoses. However, methodological improvements are needed in order to provide reliable diagnostic and prognostic information.

## Figures and Tables

**Table 1:** Patient characteristics and methylation profiles.

Case	Sex	Age	Localization	SRG	TTR in Months	Classified as Meningioma	Subclassification (Cut-Off 0.9)	Subclassification (Cut-Off 0.6)
Group I								
1	F	71	Convexity	3	6.1	Match	No match	Intermediate-A
2	F	75	Convexity	2	0.7	Match	No match	Intermediate-A
3	F	72	Basal	2	20.5	Match	No match	Intermediate-A
4	M	65	Falcine	2	56.5	Match	No match	No Match
5	F	72	Convexity	2	0.1 *	No match	No match	Benign-1
6	F	49	Convexity	2	42.0	Match	Benign-1	
7	M	47	Fossa posterior and tentorial	3	5.6	Match	No match	Intermediate-A
8	M	86	Convexity	1	18.9	Match	No match	Benign-1
9	F	36	Convexity	1	17.6	Match	No match	Intermediate-A
10	F	54	Convexity	1	15.0	Match	No match	Malignant
Group II								
11	F	42	Convexity	2	-	Match	No match	No match
12	F	60	Convexity	1	-	Match	No match	Benign-2
13	F	67	Convexity	2	-	No match	No match	Benign-2
14	F	65	Convexity	1	-	Match	No match	Benign-1
15	F	66	Convexity	2	-	Match	No match	No match
16	F	46	Convexity	1	-	Match	Benign-1	
17	M	83	Convexity	2	-	Match	Intermediate-A	
18	F	80	Basal	2	-	Match	No match	Intermediate-A
19	M	47	Convexity	1	-	Match	Intermediate-B	
20	M	38	Convexity	1	-	Match	No match	Benign-2

F; Female; M; Male; SRG; Simpson Resection Grade; TTR; Time to recurrence. Subclassifications (Benign-1, Benign-2, Intermediate A, Intermediate B and Malignant) are described in the study of Sahm et al. [17]. * Peri-operative death.

**Table 2:** *NF1/2* gene mutation analyses.

Case	*NF1/NF2* Status	*NF1/NF2* Mutation; DNA (Protein)	22q Loss
Group I			
1	No mutations identified		+
2	*NF2* mutation ^2^	c.1048delG, p.(Glu350AsnfsTer14)	+
3	*NF1* mutation ^1^	c.8301_8302delGAinsTT, p.(Gln2767_Ser2768delinsHisCys)	+
4	No mutations identified		+
5	*NF2* mutation ^2^	c.432C > G, p.(Tyr144Ter)	+
6	*NF2* mutation ^2^	c.114 + 3A > C, p.(?) #	+
7	*NF2* mutation ^2^	c.837_838delAA, p.(Lys279AsnfsTer14)	+
8	*NF2* mutation ^2^	c.467_473delGTGTTCA, p.(Ser156ThrfsTer16)	+
9	*NF2* mutation ^2^	c.297delA, p.(Lys99AsnfsTer24)	+
10	*NF2* mutation ^2^	c.1093G > T, p.(Glu365Ter)	+
Group II			
11	*NF1* mutation ^1^	c.3336C > A, p.(Asn1112Lys)	+
12	No mutations identified		
13	No mutations identified		
14	*NF2* mutation ^2^	c.543_544delGGinsTT, p.(Glu182Ter)	+
15	*NF2* mutation ^2^	c.1575-6C > A, p.(?)¤	+
16	No mutations identified		+
17	*NF2* mutation ^2^	c.448-1G > C, p.(?)	+
18	No mutations identified		+
19	*NF2* mutation ^2^	c.1396C > T, p.(Arg466Ter)	+
20	No mutations identified		

Transcript: ^1^: NM_001042492.2; ^2^: NM_000268.3, #: Predicted to destroy donor splice site (−93% by MaxEntScan, NNSPLICE and SpliceSiteFinder-like). ¤: Predicted to introduce new stronger acceptor splice site, leading to inclusion of 4 intronic nucleotides and thereby frameshift (−98% by MaxEntScan, NNSPLICE and SpliceSiteFinder-like); +: chromosomal loss of chromosome 22q present.

## Data Availability

The datasets generated during and/or analyzed during the current study are available from the corresponding author on reasonable request.

## Supplementary Materials

The following are available online at https://www.mdpi.com/2072-6694/13/6/1262/s1, Supplementary files are given in separate Microsoft Excel files. In the supplementary files complete data of all cases with associated details are included.
